# An economic analysis of premarriage prevention of hepatitis B transmission in Iran

**DOI:** 10.1186/1471-2334-4-31

**Published:** 2004-09-04

**Authors:** Peyman Adibi, Mohammadreza Rezailashkajani, Delnaz Roshandel, Negar Behrouz, Shahin Ansari, Mohammad Hossein Somi, Saeed Shahraz, Mohammad Reza Zali

**Affiliations:** 1 Research Center for Gastroenterology and Liver Disease, Shaheed Beheshti University of Medical Sciences, Tehran, Iran

## Abstract

**Background:**

To assess the economic aspects of HBV (hepatitis B virus) transmission prevention for premarriage individuals in a country with cultural backgrounds like Iran and intermediate endemicity of HBV infection.

**Methods:**

A cost-effectiveness analysis model was used from the health care system and society perspectives. The effectiveness was defined as the number of chronic HBV infections averted owing to one of the following strategies:

**1)** HBsAg screening to find those would-be couples one of whom is HBsAg positive and putting seronegative subjects on a protection protocol comprising HBV vaccination, single dose HBIG and condom protection.

**2)** HBsAg screening as above, in addition to performing HBcAb screening in the HBsAg negative spouses of the HBsAg positive persons and giving the protocol only to HBcAb negative ones.

Sensitivity and threshold analyses were conducted.

**Results:**

The cost of each chronic infection averted was 202$ and 197$ for the strategies 1 and 2, respectively. Sensitivity analysis showed that strategy 2 was always slightly cheaper than strategy 1. The discounted threshold value for the lifetime costs of chronic liver disease, above which the model was cost saving was 2818$ in strategy 1 and 2747$ in strategy 2.

**Conclusions:**

Though premarriage prevention of HBV transmission in the countries with cultural backgrounds similar to Iran seems cost saving, further studies determining precise costs of HBV infection in Iran can lead to a better analysis.

## Background

Hepatitis B is an important health problem and a major cause of acute and chronic hepatitis, cirrhosis and hepatocellular carcinoma. Approximately 30% of the world population (1.8 billion people) have the serologic evidence of HBV infection of whom 350 million are estimated to suffer from chronic HBV (hepatitis B virus) infection; at least 500,000 chronically infected people die of liver malignancy and cirrhosis each year [1].

According to Iranian studies, about 22% to 37% of general population in Iran are HBcAb positive [2,3] (e.g. previous exposure to HBV) and about 1.3% to 8.69% of the population are chronic HBV carriers [2-6]. Compared to the United States where HBV is the cause of 25% of chronic hepatitis cases, HBV accounts for up to 70% to 80% of chronic hepatitis cases in Iran [7]. Therefore, HBV alone is the leading cause of chronic liver disease (CLD) in Iran and it is evident that HBV transmission prevention can be one of the health priorities in the country.

We believe that major routes of HBV transmission, local epidemiological factors and the already performed prevention programs of each region are among important factors in identifying the populations at risk of the infection and planning the region-specific prevention strategies. In Iran, universal neonatal vaccination against HBV started in 1993 according to WHO recommendations. It means that Iranians 10 years of age or older at the time of this study received no prevention services against HBV and so most of them could contract the infection if attacked by the virus. For this at-risk population, several preventive strategies can be suggested which of course should have economic justification. Premarriage transmission prevention can be considered as one of the possible solutions to protect this population though it should not be regarded as the only or the best solution available.

Sexual contact is one of the common routes of HBV transmission. In Iran, due to a particular cultural and religious background, homosexuality is not known as a common phenomenon compared to the western countries. For the same reasons, it is very unlikely for an individual to have sexual contact (especially in the form of intercourse) with his/her would-be spouse. On the other hand, almost all premarriage individuals (those considering legal marriage) are obliged by Iranian law to undergo a predefined battery of screening tests in government-designated laboratories; this can make premarriage individuals an accessible group for a preventive intervention. Finally, since most premarriage individuals are in young age groups, they have a rather long life expectancy, which allows enough time for them to suffer from chronic complications of HBV infection in productive years of life.

We decided to perform this study to provide health policy makers in Iran and those countries with similar demographic conditions (especially in the Middle East) with an economic analysis of premarriage prevention of hepatitis B transmission.

## Methods

### Model

**Figure 1:**
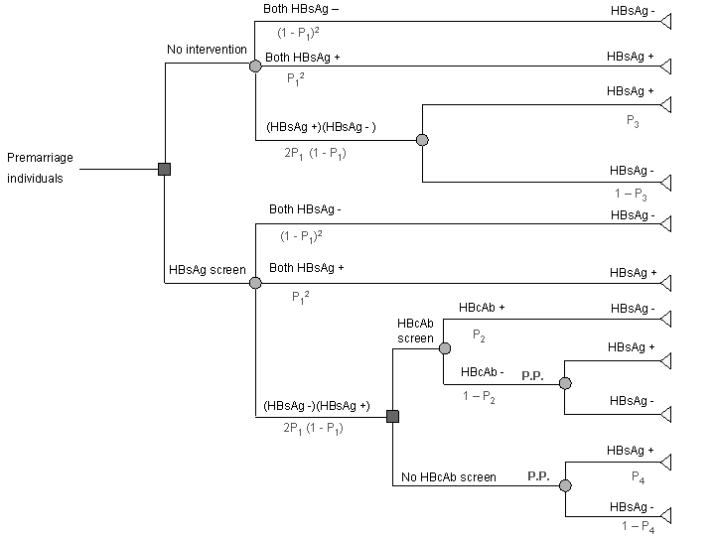
The decision tree model for premarriage prevention of HBV sexual transmission. Two similar intervention strategies start with HBsAg screening of all premarriage individuals. HBsAg negative individuals whose would-be spouse is HBsAg positive are considered for further intervention. Strategy 2 contains additional screening for HBcAb and applying the preventive protocol (P.P.) -see text- only to those negative for HBcAb (assumed to be serosusceptible) whereas in strategy 1, no HBcAb screening occurs and the prevention protocol (P.P.) is immediately performed. Only important probabilities are depicted in the figure. P_1_, P_2_, P_3_, P_4_ are defined in Table 1. P.P. stands for the preventive protocol explained in methods section in text.

The economics of performing two rather similar strategies in addition to no intervention strategy were compared using a decision tree (Fig. 1). The software used for analysis was Decision Analysis by TreeAge (DATA™, Williamstown, MA, USA).

As shown in Fig. 1, the overall options available for premarriage individuals can be one of the major strategies mentioned below:

1. Screening all premarriage individuals for HBsAg and then performing the following prevention protocol (marked as P.P. in Fig. 1) for HBsAg negative individuals whose would-be spouse is HBsAg positive:

**a.** Three-dose HB vaccine (0, 1, 6 mo)

**b.** Single dose HBIG injection

**c.** Using condoms (2 boxes/mo) during all intercourses for 7 months

**d.** Measurement of HBsAb (hepatitis B surface antibody) 1 month after the 3rd dose of the vaccine

**e.** An extra dose vaccine and additional condom protection for another month for the persons whose HBsAb is not in protective ranges (lower than 10 IU/l)

No protection protocol is considered for those couples who are both HBsAg positive or negative.

2. Screening all premarriage individuals for HBsAg followed by rescreening of the HBsAg negative spouses of HBsAg positive persons for HBcAb and finally performing the prevention protocol (e.g. the **a to e** steps above) only for HBcAb (and HBsAg) negative individuals whose would-be spouse is HBsAg positive.

No protection protocol is considered for those couples who are both HBsAg positive or negative.

3. No screening and no prevention.

The main analysis considered the perspective of health care system. However, in the final analysis (including the sensitivity analysis), a threshold analysis was performed from societal perspective. The results were expressed by the cost per chronic infection (e.g. more than 6 months HBsAg positive) averted (e.g. average cost effectiveness).

### Assumptions

The assumptions in the model included:

1) HBV vaccination is not harmful for the individual or community;

2) the efficacy of preventive methods such as 3-dose HB vaccine, HBIG injection and condoms in preventing sexual transmission of HBV do not significantly vary by geographic region;

3) the compliance of the population for receiving the preventive methods is 100%;

4) the preventive methods are available throughout the country and can cover 100% of the population;

5) HBcAb positive persons are not at risk of HBV infection and this group will not be considered for receiving preventive measures;

6) all HBsAg positive individuals are HBcAb positive too;

7) the sensitivity and specificity of the screening tests (for HBsAg and HBcAb) are 100%;

8) the average age at marriage is 25 years of age for both sexes;

9) the costs associated with CLD are incurred during a 10-year period starting at the age of 50 [1,24];

10) the transmission rate of HBV from men to women and vice versa are equal;

11) premarriage individuals do not have intercourse before marriage; and

12) Iran is located in a region with intermediate prevalence of hepatitis B.

### Probabilities and costs

The probabilities included in the decision analysis model (Table 1) were assumed to be of 2 types: 1) the probabilities that do not significantly vary by geographic location (e.g. the efficacy of 3-dose HB vaccine, HBIG injection and condom in preventing sexual transmission of HBV) and 2) the ones that seem to be significantly different in various geographic locations (e.g. HBsAg or HBcAb prevalence rates in the society).

To have preliminary estimates of the latter category probabilities, all available and accurate Iranian medical literature (published from 1993 to 2003) were reviewed. For the former category probabilities, international resources (PubMed) were included in addition to the Iranian sources mentioned above. If a probability was not found in medical literature, the consensus of an expert team including 5 gastroenterologists collaborating with our research center was considered as the base case value.

A review of Iranian studies showed that the prevalence of HBsAg in general population varied from 1.2% to 8.69% in different parts of Iran [2-6]. In this study, we considered an average rate of 2% as the baseline in a range of 1% to 9% (Table 1). The Iranian studies also showed that the prevalence of HBcAb varied from 15% to 37% in different parts of Iran. Therefore, we assumed an average rate of 20% as the baseline in a range of 15% to 40% (Table 1) [2,3].

The review of international and Iranian medical literature revealed that the probability of being HBsAg positive for an HBsAg positive person's spouse (P3) is about 4% to 15% [8-11]. We assumed a baseline probability of 5%. (Table 1)

The probability of becoming HBsAg positive for an HBsAg positive person's spouse after receiving the prevention protocol (P4) was indirectly calculated by the formula below:

P_4_ = P_3_ (1 - efficacy of preventive protocol)

The baseline value for the efficacy of the prevention protocol used in our model to protect against spouse-spouse HBV transmission (3-dose HB vaccine, HBIG injection and condom protection up to complete immunity) was assumed to be 90%. The figure was reached by considering the values found in the literature for the efficacy of 3-dose HB vaccine [12,13], HBIG injection [14-19] and condom protection [20-23] which was finally modified by consensus from the expert team described above. The most pessimistic and optimistic estimations for the efficacy of the prevention protocol were assumed to be 75% and 100%, respectively. Therefore, the baseline value for P_4_ was assumed to be 0.05% in a range of 0% to 1.25%.

The direct medical costs of interventions (Table 2) were extracted from the resources and tariffs of Iranian Health Ministry, Iran Pasteur Institute and Iranian Transfusion Organization in 2003 (unpublished data) and were used as baseline costs in the model. The indirect medical costs of the intervention such as transportation and time costs for the recipients of the preventive methods (to receive the services) were assumed to be zero and were not included in the model. The costs of the averted morbidity (HBV infection especially chronic liver disease) were not directly put in the model because of unavailability of relative Iranian studies. However, as a solution to better analysis, the latter costs were calculated as a final variable in a threshold analysis, considering the fact that of the adults in chronic carrier state, 15% will eventually develop chronic liver disease (CLD) [1,24,25]. In most economic analyses, the costs are modified for the outcomes occurring in the future, a process called discounting. Considering the average age at marriage and the age at which CLD starts (see assumptions) the cost of CLD calculated through the threshold analysis was discounted by a discount rate of 3% to the beginning of the 10-year period of CLD development. The costs are expressed in the text and tables in US $ and Iranian Rials (1US $ = 8300 Iranian Rials). The currency conversion rate reported here is the one for mid-2003 when the study was performed.

### Sensitivity analysis

Uncertainty management, which is one of the central processes in decision-making, usually involves working with probabilities that usually vary in different circumstances. Therefore, the outcome values and final decision is prone to change when the value of probabilities change. Sensitivity analysis is a method in which the final decision and the value of outcomes are estimated while each probability (univariate) or combinations of probabilities (multivariate) are varied in a reasonable range. This will reveal the variables whose change the model is sensitive to.

In this study, univariate sensitivity analysis was performed for prevalence of HBsAg positivity in general population (P_1_), prevalence of HBcAb positivity in general population (P_2_), probability of becoming HBsAg positive for an HBsAg positive person's spouse (P_3_) and probability of becoming HBsAg positive for an HBsAg positive person's spouse after receiving the prevention protocol (P_4_). Multivariate sensitivity analysis was performed for P_1_ and P_2_, considering the fact that the two probabilities were dependent. All of the direct medical costs of the intervention (Table 2) were assumed to be constant in the sensitivity analyses.

The results of the above sensitivity analyses were evaluated in respect of their impact on the value of the cost per chronic HBV infection averted and the preference of strategies 1 and 2.

## Results

Having run the model for baseline values (Tables 1 and 2), the average cost effectiveness of strategies 1 (without additional screening for HBcAb) and 2 (including additional screening for HBcAb) were 1,675,500 Rials (202 $) and 1,633,200 Rials (197 $) for each chronic HBV infection prevented, respectively.

The worst-case analysis (e.g. setting all input probability values so that they would act to decrease effectiveness and increase the costs) was performed setting P_1_, P_2_ and P_3_ at their minimum values and P_4_ at its maximum value. It showed that the average cost-effectiveness ratio of strategy 1 would be 2,460,204 Rials (296 $) and that of strategy 2 would be 2,440,015 Rials (293 $) in the worst case. It is noteworthy that the costs were not varied and were kept at their baseline values in this sensitivity analysis.

A threshold analysis was performed to find the threshold value for the lifetime cost of CLD for one individual (from a societal perspective) below which the interventions in the model were not cost saving (e.g. the net benefit was negative). The analysis was performed keeping all other variables at their baseline values. The preliminary (non-discounted) threshold value for the lifetime cost of CLD was found to be 11,170,000 Rials (1346 $) and 10,888,000 Rials (1312 $) using strategies 1 and 2, respectively. After discounting, the threshold figures for CLD costs were 23387500 Rials (2818 $) for strategy 1 and 22797054 Rials (2747 $) for strategy 2. This showed that for the cost of CLD higher than the thresholds above, the respective strategies used for HBV transmission prevention would be cost saving.

### Sensitivity analysis

**Figure 2:**
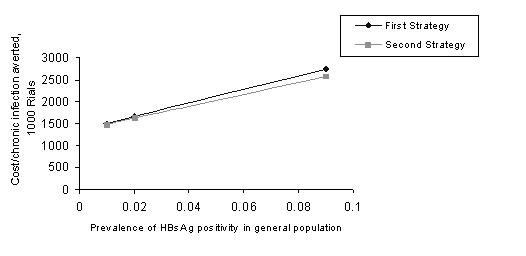
While the prevalence of HBsAg positivity in general population (P_1_) varied from the minimum to maximum in sensitivity analysis, the cost per HBV chronic infection averted varied from 1,503,440 Rials (183 $) to 2,740,560 Rials (330 $) in strategy 1 and from 1,482,620 Rials (179 $) to 2,568,310 Rials (309 $) in strategy 2; strategy 2 remained slightly cheaper throughout the range.

Sensitivity analysis showed that when the prevalence of HBsAg positivity in general population (P_1_) varied from the minimum to maximum, the cost per chronic HBV infection averted varied from 1,503,440 Rials (183 $) to 2,740,560 Rials (330 $) in strategy 1 and from 1,482,620 Rials (179 $) to 2,568,310 Rials (309 $) in strategy 2 (Fig. 2).

**Figure 3:**
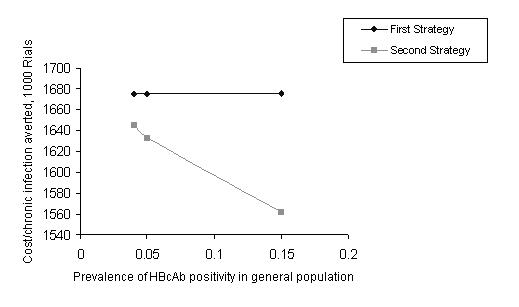
When the prevalence of HBcAb positivity in general population (P2) increased from the lowest to highest limit, the cost per chronic HBV infection prevented did not vary in strategy 1, but in strategy 2, it decreased from 1,645,720 Rials (198 $) to 1,561,530 Rials (188 $). Therefore, the higher rates of HBcAb positivity made the cost of strategy 2 become more remarkably lower than that of strategy 1.

When the prevalence of HBcAb positivity in general population (P_2_) increased from the lowest to highest, the cost per chronic HBV infection prevented did not vary in strategy 1; but in strategy 2, it decreased from 1,645,720 Rials (198 $) to 1,561,530 Rials (188 $). Therefore, the higher rates of HBcAb positivity made the cost of strategy 2 become remarkably lower than that of strategy 1 (Fig. 3).

**Figure 4:**
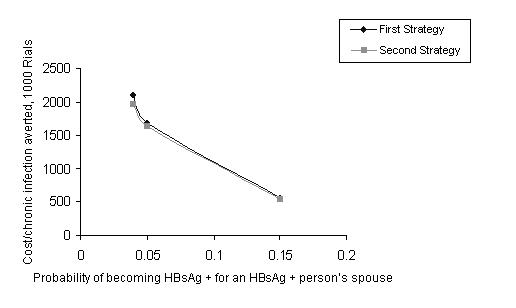
Changing the probability of becoming HBsAg positive for an HBsAg positive person's spouse (P3) after marriage from minimum to maximum varied the cost per HBV chronic infection averted from 2094370 Rials (252 $) to 558,500 Rials (67 $) in strategy 1 and from 2,041,500 Rials (246 $) to 544,400 Rials (66 $) in strategy 2. It shows that higher spouse-to-spouse transmission rates significantly increase the cost of both strategies.

Changing the probability of becoming HBsAg positive for an HBsAg positive person's spouse after marriage (P_3_) from minimum to maximum varied the cost-effectiveness ratio from 2094370 Rials (252 $) to 558,500 Rials (67 $) in strategy 1 and from 2,041,500 Rials (246 $) to 544,400 Rials (66 $) in strategy 2. It shows that higher spouse-to-spouse transmission rates significantly increase the cost of both strategies (Fig 4).

**Figure 5:**
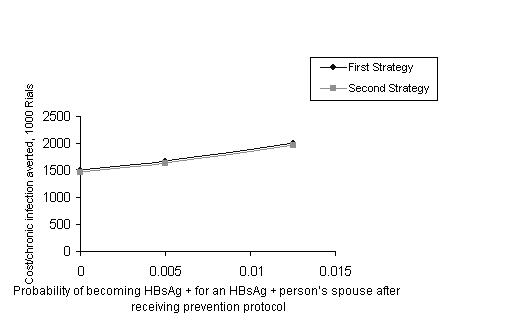
When the probability of becoming HBsAg positive for an HBsAg positive person's spouse after receiving prevention methods (P4) changed from the highest to lowest, the cost per HBV chronic infection averted decreased from 2,010,600 Rials (242 $) to 1,507,950 Rials (182 $) in strategy 1 from 1,959,840 Rials (236 $) to 1,469,880 Rials (177 $) in strategy 2. It shows that higher efficacy of the preventive intervention results in lower costs-effectiveness ratios.

When the probability of becoming HBsAg positive for an HBsAg positive person's spouse after receiving prevention protocol (P_4_) changed from *the highest to lowest*, the cost-effectiveness ratio decreased from 2,010,600 Rials (242 $) to 1,507,950 Rials (182 $) in strategy 1 and from 1,959,840 Rials (236 $) to 1,469,880 Rials (177 $) in strategy 2. It shows that higher efficacy of the preventive protocol results in lower costs-effectiveness ratios (Fig. 5).

Strategy 2 was always cheaper than strategy 1 for all values of P_1_, P_2_, P_3_ and P_4_ in the univariate sensitivity analyses (explained above).

The results of multivariate sensitivity analysis of the two variables P_1_(the prevalence of HBsAg positivity in population) and P_2_ (the prevalence of HBcAb positivity in population) revealed that the strategy 2 was always cheaper than strategy 1 while the two probabilities varied.

## Discussion

Preventing sexual transmission of HBV is not a new issue; however, the authors did not encounter any studies directly addressing the economic aspects of premarriage prevention of hepatitis B in their literature review. The reason can be the particular cultural backgrounds of Iranian community in which extramarital sexual relationships with the would-be spouse is expected to be rare due to strong traditional and religious bans against it. Consequently, the model used in this study may not be an appropriate one for countries with remarkable cultural difference in terms of extramarital sexual relationship such as Western countries. On the other hand, the cultural similarities between Iran and some other Eastern countries can increase the external validity of our model for policy makers in such countries.

One of the important challenges in our study was the lack of precise data regarding some probabilities. Most of the studies in our literature review contained the prevalence of HBsAg positivity (chronic HBsAg carrier state) in the spouses of HBsAg positive people and the extent to which it was different from the rate in general population [8-11]. Because such a rate existed in literature, we preferred to use this prevalence rate representing the initial outcome of sexual contact with an HBsAg positive spouse after marriage. Thus, what is seen in the model (Fig. 1) as HBsAg+ final outcome does not mean getting infected with HBV; it means getting into a chronic HBsAg carrier state. In our model, we assumed all such cases to be chronic HBsAg carriers that entered the chronic carrier state asymptomatically or following an acute infection; 15% of such carriers could finally develop CLD during their lifespan [1,24,25]. Therefore, it is obvious that our model have primarily focused on more chronic outcomes of HBV infection (e.g. we ignored the costs of acute infections). However, this will not endanger the data robustness in our study; instead, it will always guarantee that all of the cost-effectiveness ratios calculated here are actually higher than that would be resulted with including the costs for acute cases which usually comprise a considerable portion of the symptomatic cases in adults [1,24,25].

The costs of acute and chronic liver disease (CLD) due to HBV infection in Iran were other variables for which data was lacking. One of the important reasons we did not use a Markov model to calculate CLD costs for Iran was the lack of important necessary data for running a Markov model (e.g. lack of data on age specific mortality rates, etc.). We first calculated cost-effectiveness ratios ignoring the costs of acute and chronic liver disease (the costs of the morbidity averted); this surely exaggerated the calculated cost-effectiveness ratios in the study. Thus, one should judge them from a more optimistic point of view. In the second step, to compensate for the lack of lifetime CLD costs in Iranian literature, we performed a threshold analysis using the baseline values for input probabilities and determined a threshold value for the lifetime costs of CLD in Iran, above which the preventive interventions were cost saving. The threshold levels for CLD lifetime costs estimated above do not seem high costs compared with the costs that CLD can impose on the society in terms of direct and indirect medical costs and productivity losses due to time spent sick or years of life lost because of premature death. The relevant medical costs or the costs associated with the productivity losses due to CLD was not accessible at the time of this research, so we discussed the point through some indirect comparisons considering the threshold we calculated for lifetime CLD costs.

Though comparing the costs of CLD in Iran with that in the United States does not seem a standard approach, the large differences between the threshold figure we calculated for CLD costs in Iran and the CLD costs estimations (including productivity losses) mentioned in the studies for the United States in 2001 (64,382 $) [25] may partially reveal some facts [1,24,25]. To perform a more realistic comparison, we used Purchasing Power Parity (PPP) rates instead of exchange rates to convert the threshold cost in Rials into PPP dollars. PPP is defined as the numbers of units of a country's currency needed to buy in the country the same amounts of goods and services as, say, one US dollar would buy in the United States. The PPP rate for Iranian Rial was extracted from the National Health Account 2002 by Planning and Management Organization of Iran (unpublished data) and from figures reported by the World Bank Group. The threshold cost using PPP rates would be equal to about 9615 to 9863 PPP $ (for 2 strategies). Therefore, even if the total CLD costs in a country like Iran were 7-fold smaller than that in the United States, the strategies 1 and 2 mentioned in the model could still be cost saving.

To give a sketchy view of some of CLD costs in Iran, the costs of a liver biopsy and those of a pharmacotherapy regimen related to CLD was retrieved by contacting discharge and accounting departments of a state-run hospital and a major drug distributor in Tehran. The discounted cost for an uncomplicated liver biopsy needing 1 to 2 days of hospitalization, blood coagulation serial tests and a special liver biopsy needle turned out to be about 2,093,778 Rials (252 $) in all state-run hospitals in Iran. Pharmacotherapy with new antiviral and immunomodulatory drugs is employed for treatment of active chronic liver disease in patients with hepatitis B [26]. The discounted cost of lamivudine, a typical example of these drugs, can be another instance of costs CLD imposes on many patients. A 12-month course of lamivudine (Iranian brand) in Iran can cost a patient 2,521,956 Rials (304 $). If the same calculation is performed for the foreign brand of lamivudine available in Iran (Zeffix), the discounted cost will be 12,991,892 Rials (1565 $). In addition, these costs may be much higher when some more expensive drug regimens are used or more prolonged regimens are repeated due to chronicity or intractability of disease. The costs mentioned here can not directly give a clue to total CLD costs and mentioning them was to help the reader get a view of the scale of some familiar CLD costs in Iran.

In a different approach, we converted the threshold value into the number of productive months for a national of a country having a GDP (Gross National Product) per capita like Iran. GDP per capita shows the amount that an individual contributes to domestic income every year. According to the World Bank Group, Iran had a GDP per capita of 1641 $ in 2002, and the average annual growth of GDP per capita in Iran for 2002 to 2006 was 4.5%. From here, the GDP per capita in 2003 comes to be 1790 $. Assuming that the average growth of GDP per capita for Iran will remain at 5% in the coming 25 years (the period used for discounting the CLD threshold costs), the GDP per capita will be 6062 $ at the end of the period. Considering such figures, the threshold calculated above would be equal to about 6 months of productivity based on a GDP view. The average number of Quality Adjusted Life Years (QALY) that CLD can deduct of an Iranian's life is not yet determined. Nevertheless, a 6-month period does not seem a long time compared with the life years lost due to premature mortality and the QALYs lost due to sickness in the proportion of CLD patients with cirrhosis and hepatocellular carcinoma only.

Finally, the fact that the strategies would be cost effective can be further emphasized when taking into account the costs of acute cases of HBV infection that comprise the majority of symptomatic cases in adults and were ignored in the model due to lack of Iranian data and for sake of simplicity. Another similar topic that merits discussion here is the topic of mother-to-child vertical transmission of HBV. If a female gets into a chronic HBsAg carrier state and remains positive for HBeAg during pregnancy, it is very likely that her child is infected with HBV. Since a considerable proportion of infected infants will get chronic HBV carriers, this can lead to newer CLD cases further increasing the costs of CLD. Considering this fact, the cost of averted morbidity owing to the preventive strategies will increase even more and the model would seem more cost-effective.

On the other hand, the prevention protocol in our model might seem a bit extravagantly designed when looking at the final HBsAb test and the extradose of HB vaccine administered when HBsAb serum levels are insufficient. In addition, one may argue that condoms may be more available while being nearly as protective against HBV transmission as HBIG injection. This can be useful in modifications poor countries can make to the model to get similar results in lower price or with more flexible/available choices.

The average cost-effectiveness ratios associated with the two preventive strategies shown in the model did not differ much. The strategy 2 was always slightly cheaper than strategy 1. When the prevalence of HBcAb positive people in the general population (P2) rose, strategy 2 would get remarkably cheaper.

Finally, it is noteworthy to mention the issue of compliance. As explained in methods, we assumed all recipients of the preventive interventions (strategies 1 and 2) would be 100% compliant and there was a full coverage of such services in the country. The reason for such assumption was the strict regulations set by Iranian government for all premarriage individuals to undergo a battery of screening tests in which the strategies in our model could be integrated. Nevertheless, we can assume that compliance variations can affect the efficacy of our prevention protocol (e.g. with lower rates of compliance for accepting preventive strategies, we will have lower efficacy of the prevention protocol). As stated in results, we performed sensitivity analysis for P4 (probability of becoming HBsAg positive for an HBsAg positive person's spouse after receiving the prevention protocol), which is a variable dependent on the efficacy of the prevention protocol (see the formula in methods). Therefore, we indirectly incorporated compliance into our model's sensitivity analysis. Considering compliance issue, we may prefer strategy 1 because it includes fewer steps (e.g. it does not include screening for HBcAb) and may be easier to administer in a low compliance population especially that it is negligibly more expensive than strategy 2.

## Conclusions

Finally, we conclude that applying the preventive strategies in our model for HBVsexual transmission prevention before marriage in the countries with cultural backgrounds similar to Iran seems cost saving. Further investigations in the country for precise calculation of costs of HBV infection especially the costs associated with CLD is necessary for more accurate economic evaluations.

## Competing Interests

Nond declared.

## Author's Contributions

**PA**: Proposing the main idea, supervising the project and counseling the methodology development

**MR**: Developing methodology, critical appraisal, rewriting the final article from draft, literature review, and responding to reviewers

**DR**: Literature review, methodology development, decision tree development, calculations and results, and writing the first draft of the article

**NB**: Contributing to decision tree development, searching literature for costs

**SA**: Review literature for probabilities, contributing to decision tree

**MHS**: Counseling the scenarios of the decision tree model

**SS**: Counseling the methodology, model development and discounting problems

**MRZ**: Senior supervisor of the research project

All authors read and approved the final manuscript.

## Pre-publication history

The pre-publication history for this paper can be accessed here:



## Figures and Tables

**Table 1:** Input values for the probabilities in the model. Min. and Max. values were used in sensitivity analysis.

**Variable**	**Definition**	**Baseline**	**Min.**	**Max.**	**References**
P_1_	Prevalence of HBsAg positivity in general population	0.02	0.01	0.09	2–6
P_2_	Prevalence of HBcAb positivity in general population	0.20	0.15	0.40	2,3
P_3_	Probability of becoming HBsAg + for an HBsAg + person's spouse	0.05	0.04	0.15	8–11
P_4_	Probability of becoming HBsAg + for an HBsAg + person's spouse after receiving prevention protocol ^§^	0.005	0	0.0125	12–23

^§^ See text for details.

**Table 2:** Input values for the costs in the model.^§^

**Variable**	**Definition**	Cost Rials (US $)*
C1	Cost of HBsAg screening	30000 (3.6)
C2	Cost of HBcAb screening	30000 (3.6)
C3	Cost of HB vaccination (one dose)	40000 (4.8)
C4	Cost of HBIG injection (single dose)	120000 (14.5)
C5	Cost of Condoms (one box)	8000 (1)
C6	Cost of HBsAb screening	30000 (3.6)

^§^ The direct medical costs of the interventions were extracted from the resources and tariffs of Iranian Health Ministry, Iran Pasteur Institute and Iranian Transfusion Organization (unpublished data).

* 1US $ = 8300 Iranian Rials
